# Properties of the Nucleo-Olivary Pathway: An *In Vivo* Whole-Cell Patch Clamp Study

**DOI:** 10.1371/journal.pone.0046360

**Published:** 2012-09-27

**Authors:** Paolo Bazzigaluppi, Tom Ruigrok, Payam Saisan, Chris I. De Zeeuw, Marcel de Jeu

**Affiliations:** 1 Department of Neuroscience, Erasmus Medical Center Rotterdam, Rotterdam, The Netherlands; 2 Department of Neuroscience, University of California San Diego, La Jolla, California, United States of America; 3 Netherlands Institute for Neuroscience, Amsterdam, The Netherlands; University of Southern California, United States of America

## Abstract

The inferior olivary nucleus (IO) forms the gateway to the cerebellar cortex and receives feedback information from the cerebellar nuclei (CN), thereby occupying a central position in the olivo-cerebellar loop. Here, we investigated the feedback input from the CN to the IO *in vivo* in mice using the whole-cell patch-clamp technique. This approach allows us to study how the CN-feedback input is integrated with the activity of olivary neurons, while the olivo-cerebellar system and its connections are intact. Our results show how IO neurons respond to CN stimulation sequentially with: *i)* a short depolarization (EPSP), *ii)* a hyperpolarization (IPSP) and *iii)* a rebound depolarization. The latter two phenomena can also be evoked without the EPSPs. The IPSP is sensitive to a GABA_A_ receptor blocker. The IPSP suppresses suprathreshold and subthreshold activity and is generated mainly by activation of the GABA_A_ receptors. The rebound depolarization re-initiates and temporarily phase locks the subthreshold oscillations. Lack of electrotonical coupling does not affect the IPSP of individual olivary neurons, nor the sensitivity of its GABA_A_ receptors to blockers. The GABAergic feedback input from the CN does not only temporarily block the transmission of signals through the IO, it also isolates neurons from the network by shunting the junction current and re-initiates the temporal pattern after a fixed time point. These data suggest that the IO not only functions as a cerebellar controlled gating device, but also operates as a pattern generator for controlling motor timing and/or learning.

## Introduction

**Figure 1:**
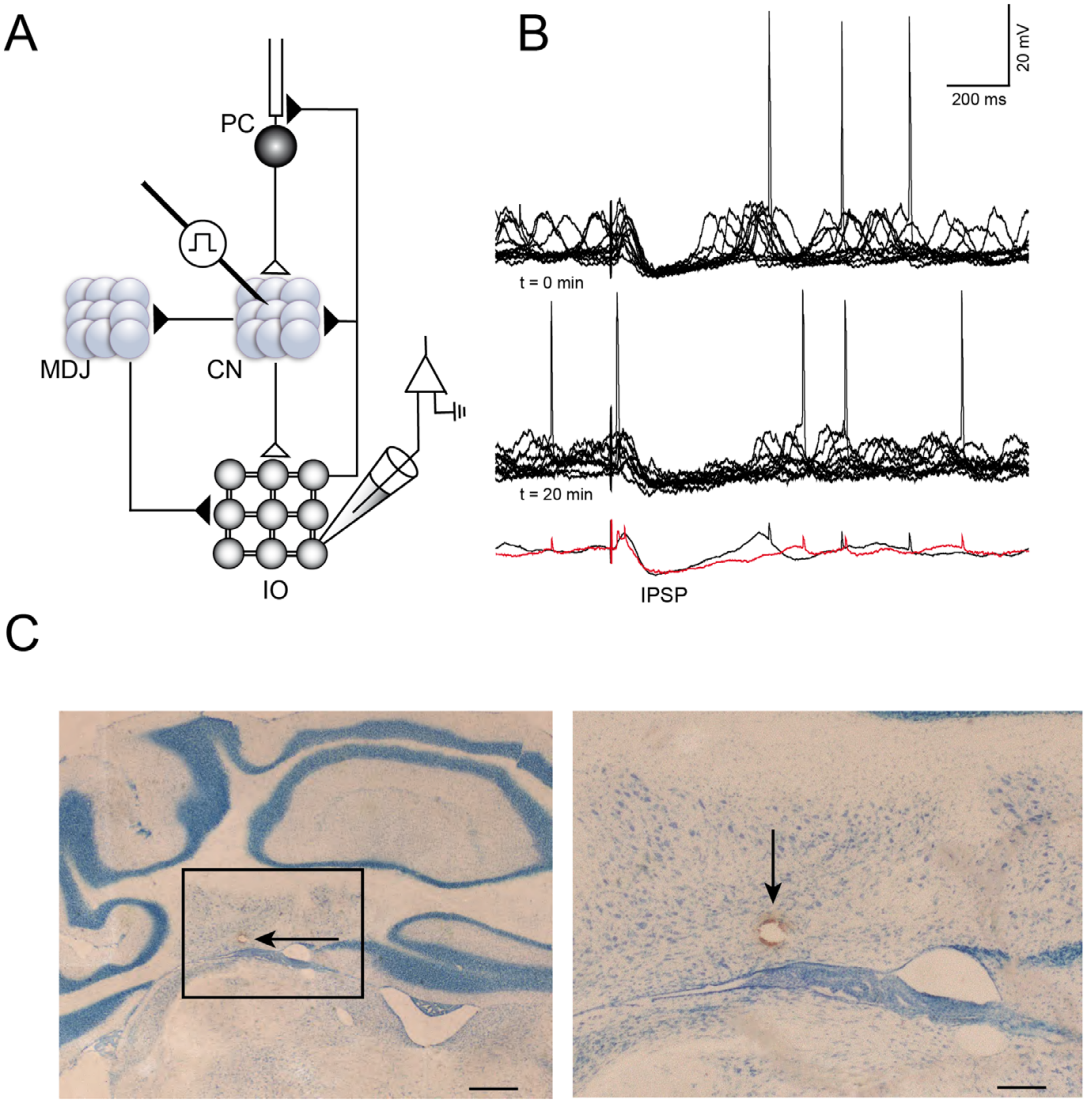
*In vivo* CN-evoked IO response. A: experimental set-up: the stimulation electrode is placed in CN, the recording pipette is in the IO. Synapse, closed triangle: excitatory, open triangle: inhibitory. B: control experiment, example of LTO cell responding to CN stimulation, top trace: beginning of the experiment (t = 0); middle trace: after twenty minutes (t = 20), bottom trace: averages of the two conditions above, black trace is t = 0; red trace is t = 20. There are no significant changes. C: coronal sections of cerebellum. Left: 1.6 magnifications, arrow points at the lesion in the Interpositus Nucleus, scale bar: 0.5 mm. Right: same as left, 4.6 magnifications, arrow points at the lesion, scale bar: 0.15 mm. Abbreviations: CN, Cerebellar Nuclei; MDJ, Meso-diencephalic Junction; PC, Purkinje Cell; IO, Inferior Olive.

The inferior olive (IO) is located in the ventral medulla and gives rise to the climbing fibres (CFs), which constitute one of the two main excitatory inputs to the Purkinje cells (PCs) in the cerebellar cortex. Olivary neurons, which are coupled via dendro-dendritic gap junction (GJ) [1], also send off collaterals to the cerebellar nuclei (CN). PCs send inhibitory fibres to the CN, which contain GABAergic, glycinergic and glutamatergic neurons. Part of the CN neurons projects directly to the IO via an inhibitory, GABAergic, pathway [2]–[6]; whereas another population of CN neurons excites the IO indirectly via nuclei located at the mesodiencephalic junction (MDJ) [7], [8]. This olivo-cortico-nuclear projection (Figure 1A) forms the basis of the modular organization of the cerebellum [9]. Despite our anatomical knowledge on this nucleo-olivary projection, its role in motor control and motor learning is still under debate [10], [11]. The GABAergic feedback inhibition on the IO might serve to gate motor learning in the cerebellar cortex [2], [12] or to control the participation of IO neurons in a motor task by controlling the electrical coupling between olivary neurons [13]–[15].

Although the anatomical evidence of GABAergic inputs being present in the IO is overwhelming [3], [7], [16], electrophysiological experiments, surprisingly, failed to reveal the presence of spontaneous inhibitory potentials (IPSPs) when olivary neurons’ activity was recorded *in vitro* [14], [17]–[20] or *in vivo* [21]–[23]. The only two *in vitro* studies in which olivary GABAergic IPSPs were observed [24], [25], were performed under experimental conditions specifically designed to solely observe the GABAergic response. Both studies are important because they directly show the actual presence and the activation of GABA_A_ receptors on the membrane of IO neurons. Devor et al. [25] showed additionally that there is a differential distribution of GABA_A_ receptor subtypes between the dendrites and soma of IO neurons and Best and Regehr [24] showed that the release of GABA is exclusively asynchronous and that the synaptic transmission was extremely frequency dependent, which are all important properties in order to understand the GABAergic transmission in the IO. However, both studies did not explore the contribution of this inhibitory feedback action under physiological conditions.

For these reasons, we directly activated the CN with a stimulation electrode while performing whole-cell recording from the IO *in vivo*. Our approach succeeded in evoking inhibitory responses (IPSPs) in olivary neurons and allowed us to explore their relation with the subthreshold oscillatory behaviour of the neurons. Moreover, we pharmacologically block the GABA_A_ receptors in the recorded neuron, showing the direct involvement of GABA_A_ receptors, in line with Devor et al. [25]. Ultimately, we replicated the experiments in Connexin36 knock out animals to demonstrate that the evoked GABAergic IPSPs were generated on the recorded neuron and not in the periphery of the electrotonically coupled network.

## Results

### Cerebellar Control of the Inferior Olive

**Figure 2:**
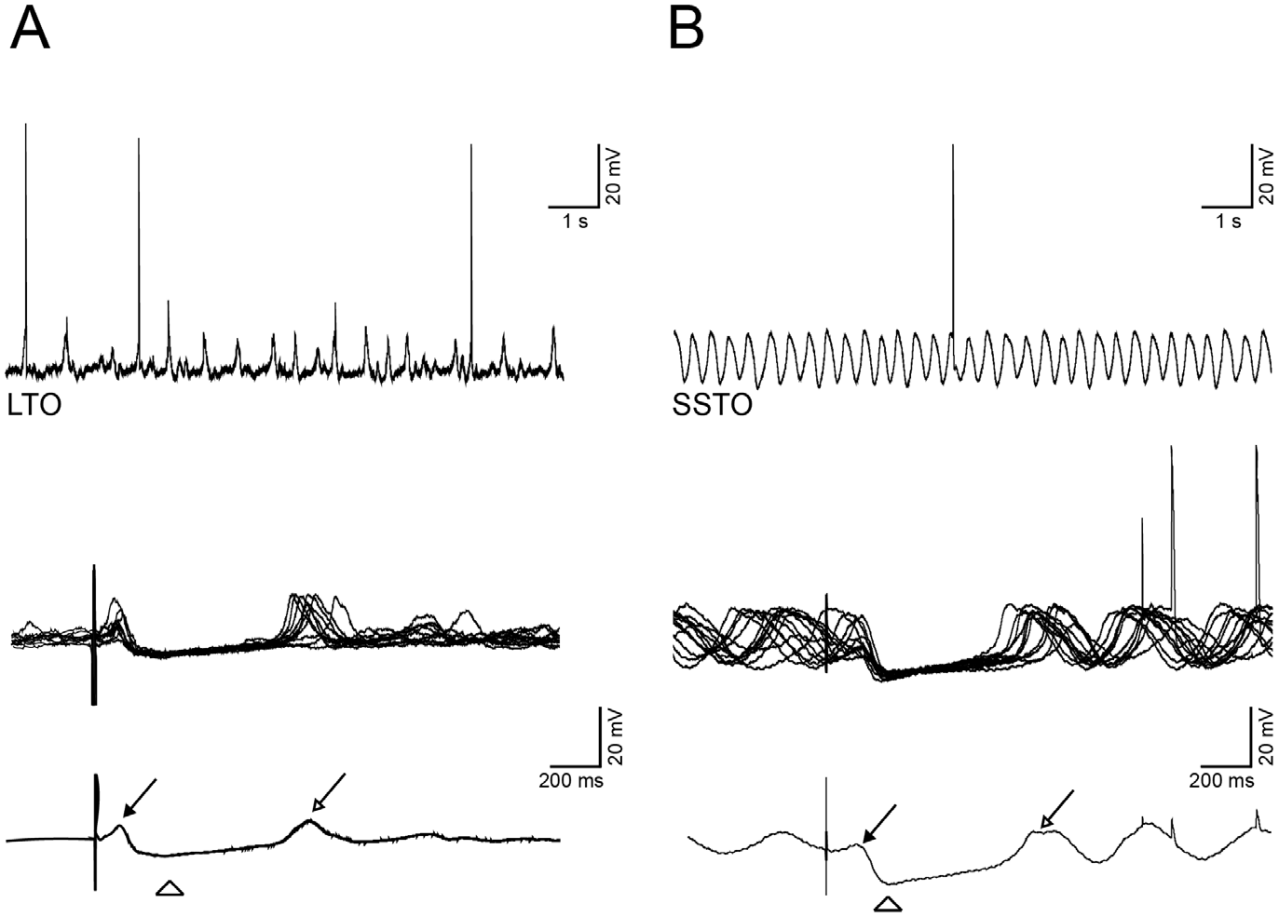
Differences in the response between LTO and SSTO neurons. A: spontaneous activity of an LTO cell (top left trace), then (middle trace) the same cell is responding to CN stimulation, bottom trace shows the average of the responses. B: same experiment but in a SSTO cell. Filled arrow: short-latency EPSP, empty arrow: rebound depolarization; empty arrowhead: peak of the IPSP.

*In vivo* whole-cell recordings allow us to monitor both intrinsic suprathreshold and subthreshold activities of olivary neurons as well as responses evoked by CN stimulation (Figure 1). The recorded neurons presented subthreshold profiles in line with the ones previously shown by Khosrovani [22]. Briefly, we focused our analysis on the IO neurons which were presenting either low-threshold oscillations (LTO) or sinusoidal subthreshold oscillations (SSTO). It is still unknown whether these two different subthreshold activities reflect two distinct neuronal populations or whether they are two different oscillating profiles of the same type of neurons. *The sinusoidal subthreshold oscillation is an intrinsic property* [*26]*–[28], *generate by a cascade of alternating channel activation* [*17*] *that includes the T-type Ca^2+^ channel Ca_V_3.1.*What emerged from our study is that both LTO and SSTO neurons responded to CN stimulation, but in a different manner. For all the neurons that were orthodromically activated, we measured the passive membrane properties, which were similar to the ones reported by Khosrovani et al. [22]: the resting membrane potential of LTO cells was −53.5±6.2 mV (n = 20), whereas SSTO neurons showed a membrane potential of −54.0±6.2 mV (n = 15). Input resistance was on average 31.3±13.7 MΩ for LTO neurons and 23.5±4.9 MΩ for SSTO, whereas membrane capacitance was 202.5±108.5 pF and 236.6±89.7 pF for LTO and SSTO neurons respectively (see Table 1). The differences between LTO and SSTO neurons with regards to their subthreshold profile are limited to the frequency, the rhythm and the shape of the oscillations as already described by Khosrovani et al. [22]. The orthodromic activation of nucleo-olivary pathway by highfrequency stimulation of the CN gave rise to different sets of inhibitory responses. The activation of the nucleo-olivary pathway resulted in a very specific olivary response pattern (Figure 1B). Both LTO and SSTO neurons are able to respond to the CN stimulation with EPSPs after a latency of 38.15 (±14.2 ms, from here on, short-latency EPSPs), although with different probability (43.1% vs. 29.1% for LTO (n = 13) and SSTO (n = 7) neurons, respectively. Table 1). The EPSP, when present, was occasionally accommodating an action potential. The long-latency IPSPs responses were consistently recorded in both LTO (n = 13) and SSTO (n = 7) neurons (88.7% and 91.5% of the cases respectively) (Table 1). The long-latency IPSP fully suppressed the generation of action potentials as well as the generation of subthreshold activity, including oscillations; however, the responses of LTO and SSTO neurons express different characteristic in this respect. The duration of the membrane hyperpolarization in LTO neurons (573±292 ms, n = 13) was significantly longer than that observed in SSTO neurons (260±58 ms, n = 7; p<0.01, t-test, Table 1, Figure 2). Moreover, the IPSPs’ peak amplitude of SSTO neurons was significantly bigger than the one of LTO neurons (−10.1±2.8 vs −6.6±1.4, p<0.01, t-test, Table 1). The long-latency IPSP responses (figure 2A and 2B) were observed with and without the preceding short-latency EPSPs, suggesting that these two responses are evoked independently from each other. We then stimulated two times in a row at different time intervals in four neurons that responded to CN stimulation with both a short-latency EPSPs and a long-latency IPSP in order to elucidate for how long the IPSP can prevent the onset of the EPSP evoked by the second stimulation. The second CN stimulation can elicit a second EPSP only if the interstimulus interval is at least 350 ms (n = 4, Fig. S1).

**Table 1:** Passive properties and sub- and supra-threshold responses to CN stimulation of LTO and SSTO olivary neurons.

	LTO	SSTO
	Mean ± SD (n)	Mean ± SD (n)
**Resting membrane potential (mV)**	−53.5±6.2 (20)	−54.0±6.2 (15)
**Input resistance (MΩ)**	31.3±13.7 (20)	23.5±4.9 (15)
**Membrane capacitance (pF)**	202.5±108.5 (20)	236.6±89.7 (15)
**Short latency EPSP (%)**	43.1 (13) ±9.2	29.1 (7) ±5.6
**Long latency IPSP (%)**	88.7 (13) ±5.6	91.5 (7) ±4.6
**IPSP duration (ms)**	573±292 (13)*	260±58 (7)*
**IPSP peak amplitude (mV)**	−6.6±1.4 (13)*	−10.1±2.8 (7)*

* indicates significant difference.

### GABAergic Inhibitions and Subthreshold Oscillations

**Figure 3:**
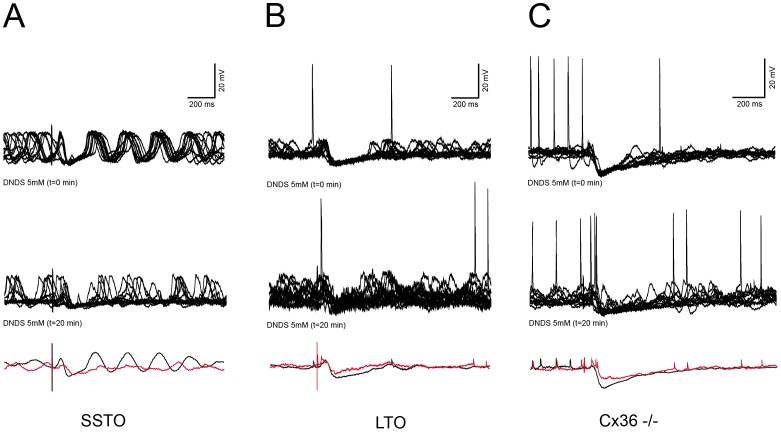
DNDS sensitivity in WT and Cx36 KO neurons. A: Wild type SSTO cell (top trace) responding to CN stimulation, then (middle trace) the response of the same cell to CN stimulation in reduced after 20 minutes of dialysis with DNDS, bottom trace represents the averages at the beginning of the experiment (black) and after 20 minutes of DNDS (red). B: same as A, but in a LTO cell. C: Cx36−/− LTO cell, the response and the DNDS sensitivity are not different from the Wild type.

The most likely candidates responsible for the hyperpolarization are the GABA_A_ receptors activated by the GABAergic pathway originating in the CN. In order to investigate the contribution of GABA in the observed long-latency hyperpolarizing responses, we added a specific GABA_A_ receptor blocker DNDS to our pipette solution [29], [30]. In order to block GABA_A_ receptors internally, DNDS molecules have to travel from the pipette to distal dendritic sites; a time consuming process of approximately 20 minutes [30]. Therefore, this experiment requires a stable recording for at least 20 minutes. A subset of the neurons presented in Table 1 was recorded long enough to explore the properties of their responses over a time span of more than 20 minutes. To quantify the hyperpolarizing response, we measured the duration, peak amplitude and surface area generated by the hyperpolarizing sag, which were not affected by the dialysis of the cytoplasm with our pipette solution (Table 2, Figure 1B). Control cells (n = 9) recorded for 20 minutes with a DNDS-free internal solution showed no significant difference in short-latency EPSPs probability, long-latency IPSPs probability, IPSPs duration, IPSP peak amplitude, IPSP area, rebound probability and amplitude between the beginning and the end of the recordings (Table 2). On the other hand, the presence of DNDS (n = 11) in the recording pipettes already affected some of the response properties that were measured immediately after breaking the membrane patch compared to the ones of control cells. The average probability, duration, surface area and peak amplitude of the IPSPs were lower, but not significantly, than the ones measured with DNDS-free solution, suggesting an immediate action of the blocker on somatic GABA_A_ receptors (unpaired t-test, for SSTO, p = 0.06, p = 0.22, p = 0.84 and p = 0.42 respectively and for LTO, p = 0.14, p = 0.17, p = 0.19 and p = 0.29 respectively, Table 2). After 20 minutes of DNDS dialysis, the chances of triggering a hyperpolarizing response was significantly reduced (LTO: 85.3 vs 25.4, n = 7, p<0.01; SSTO: 93.5 vs 39.9, n = 4, p<0.01) and so was the peak amplitude (in mV, LTO: −6.5±1.5 vs −3.4±1, n = 7, p<0.01; SSTO: −10.96±3.9 vs −4.2±1.8, n = 4, p<0.01) and the surface area of the hyperpolarization (in ms*mV, LTO: 1737±599 vs 654±150, n = 7, p<0.01; SSTO: 1718±882 vs 484±254, n = 4, p = 0.04). On the other hand the chance of triggering the short latency EPSP was not significantly altered (Table 2) and, the chance to evoke a rebound depolarization were slightly but significantly reduced only in the case of SSTO neurons (96.5 vs 74.2, n = 4, p<0.05). Yet, the CN stimulations can still evoke a small hyperpolarizing response after 20 minutes of DNDS dialysis, (Figure 3, Table 2). In order to exclude the putative limiting blocking effects of 5 mM DNDS, two experiments have been performed with an high DNDS concentration (15 mM), but also the higher concentration of blocker was not able to fully remove the residual hyperpolarizing response (n = 2, Fig. S2). In fact, there was no difference between the effects following 15 mM and 5 mM DNDS, the latter concentration was then assumed to be sufficient to exert a maximal effect. The residual slow hyperpolarizing component is probably due to the activation of GABA_B_ receptors which are present in the IO [31], [32]. Unfortunately, there’s no specific intracellular blocker for GABA_B_ receptors, hence it was not possible in our experimental set up to investigate the role of this metabotropic GABAergic receptor.

**Table 2:** Sub- and supra-threshold responses in Control and with presence of GABA_A_ receptors blocker (DNDS).

		Control				GABA_A_ blocker DNDS			
	Oscill. behavior	Immediately after break-in	>20 minutes after break in	p- value	n	Immediately after break-in	>20 minutes after break in	p- value	n
**Short latency EPSP (%)**	LTO	34.3±10	32.6±11.8	0.80	6	49.1±27.1	36.1±14.6	0.19	7
	SSTO	16.7±2	43.0±1.9	0.49	3	86.1±13.2	56.4±19	0.14	4
**Long latency IPSP (%)**	LTO	93.6±7.9	87.5±3.8	0.11	6	85.3±11.4	25.4±10.6	<0.01	7
	SSTO	83.8±3.9	81.9±11.2	0.88	3	93.5±4.7	39.9±14.4	<0.01	4
**IPSP duration (ms)**	LTO	512±187	518±161	0.90	6	390±26	295±14	0.02	7
	SSTO	334±179	376±138	0.38	3	219±19	198±26	0.42	4
**IPSP peak amplitude (mV)**	LTO	−7.6±2.0	−6.0±1.9	0.18	6	−6.5±1.5	−3.4±1.0	<0.01	7
	SSTO	−8.2±2.7	−6.7±1.3	0.37	3	−10.96±3.9	−4.2±1.8	<0.01	4
IPSP surface area (ms*mV)	LTO	2738±1806	2261±1268	0.57	6	1737±599	654±150	<0.01	7
	SSTO	1871±474	1896±458	0.27	3	1718±882	484±245	0.04	4
**Rebound (%)**	LTO	60.1±32	57.4±28.3	0.21	6	68.2±21.2	69±17.3	0.46	7
	SSTO	87±9.7	84.2±5.7	0.25	3	96.5±1.4	74.2±10.5	<0.05	4
**Rebound amplitude (mV)**	LTO	9.52±4.2	10.9±4	0.30	6	6.9±2.3	8.1±2.9	0.09	7
	SSTO	13.9±3.7	13.5±4.2	0.81	3	10.2±2.1	11.85±2.3	0.3	4

Olivary neurons are electrotonically coupled through dendro-dendritic Connexin36 based gap junctions. Since GABA mediated currents can be transmitted from one cell to another via these gap junctions which are also structurally related to GABAergic synapses in olivary glomeruli [16], [33], we aimed to show the importance of the electrical synapses in the expression of the CN evoked GABAergic transmission. Therefore, we replicated our experiments in mice that lack Connexin36 [23], [26], [27]. This experiment revealed that olivary responses to CN stimulation in Connexin36 knock-out mice were similar compared to those in wild type mice in that the hyperpolarizing response was also blocked by DNDS (Figure 3, Table 3). This result indicates that the olivary expression of the CN-evoked GABAergic synaptic transmission does not depend on the presence of an electrotonic network, consequently the responses we observed were generated by the activation of GABAreceptors on the primary (i.e. recorded) olivary cell and do not have a second or higher distant origin.

**Table 3:** Olivary subthreshold responses to CN stimulation in presence of GABA_A_ blocker DNDS measured in Cx-36 KO mice.

		GABA_A_ blocker DNDS			
	Oscill. behavior	Immediately after break-in	>20 minutes after break in	p- value	n
**Short latency EPSP (%)**	LTO	50.7±32.9	31.7±19.3	0.051	4
	SSTO	24.3±25.3	29.2±12.5	0.266	4
**Long latency IPSP (%)**	LTO	96.5±8	56.6±23.5	<0.05	4
	SSTO	95.8±1.3	51.8±21.8	<0.01	4
**IPSP duration (ms)**	LTO	488.7±123	335.1±36.3	0.07	4
	SSTO	373.0±199	298.2±121	0.191	4
**IPSP peak amplitude (mV)**	LTO	−8.9±4.4	−5.3±1.8	<0.05	4
	SSTO	−10.9±3	−5.5±0.6	<0.05	4
IPSP Surface area (ms*mV)	LTO	−3203.4±1775	951.5±640	<0.05	4
	SSTO	2798.1±1636.27	795.7±284.7	<0.05	4
**Rebound (%)**	LTO	50.7±13.6	58.3±18.2	0.16	4
	SSTO	77.2±15.1	63.8±20	<0.05	4

### CN Stimulation Resets Subthreshold Oscillation

**Figure 4:**
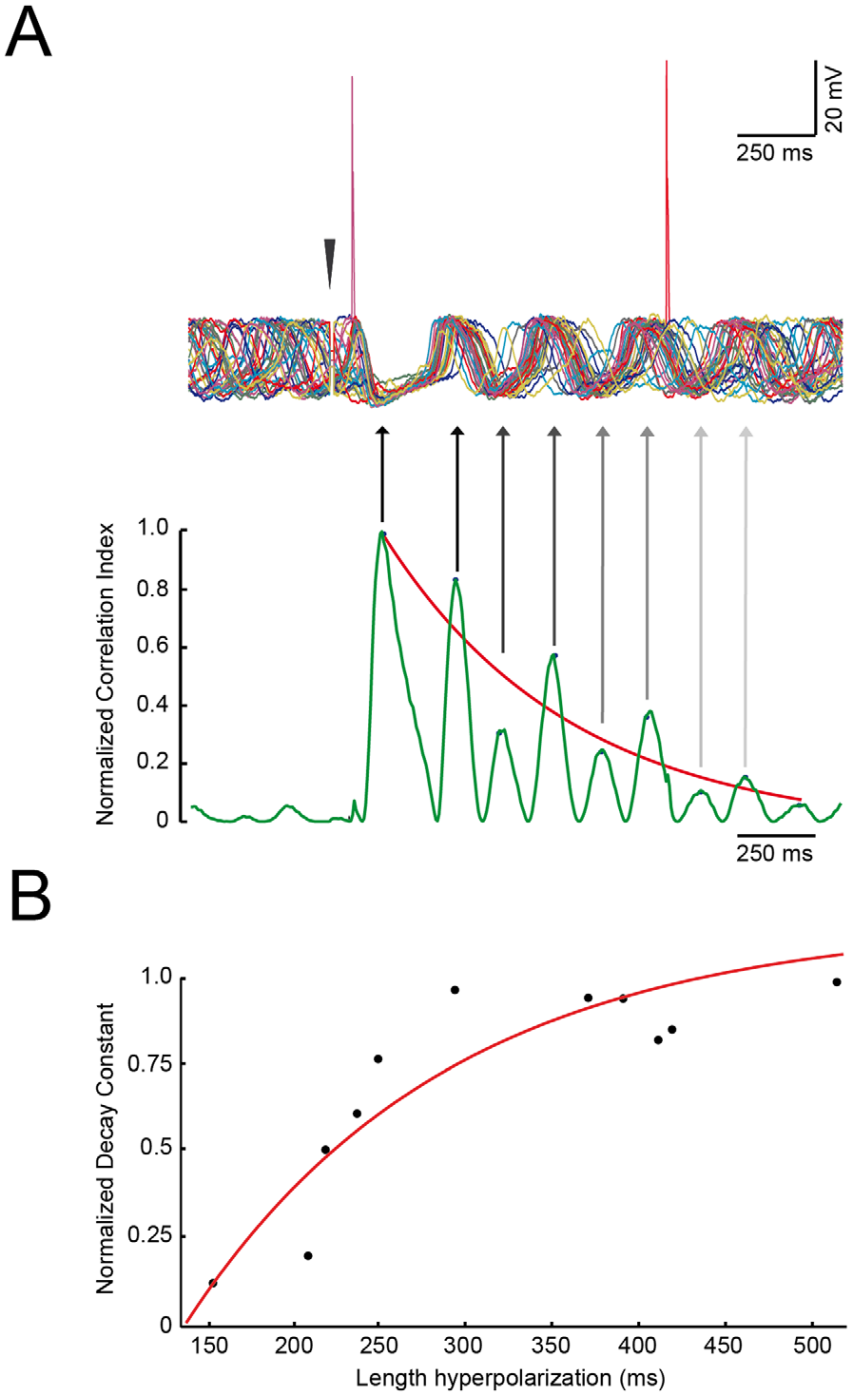
The length of the IPSP dictates the length of the phase reset. A, top: overlay of 36 random-start recording from the same unit, the stimulation artifact has been removed for clarity and substituted with a red arrowhead. A, bottom: cross-correlogram of the traces in A, top, normalized values are on the left bar. Arrows indicate the correspondence of the peak in the cross-correlogram with the rough recording. Blue dots indicate the points used for the exponential fitting that is shown as a red curve. C: the decay time of the exponential fittings plotted against the length of the hyperpolarization of each SSTO neuron are fitted with an exponential function (n = 11, r^2^ = 0.83).

The long-latency IPSPs are terminated by a rebound depolarization (Figure 2, open arrow). The rebound depolarization is related to the evoked IPSP and requires also the activation of T-type Ca^2+^ channel Ca_V_3.1 [34]–[36]. In SSTO neurons, the sinusoidal subthreshold oscillation resets after this rebound depolarization (Figure 2B and 3A). Given the fact that oscillations of LTO neurons are more arrhythmic and lack a proper phase, the reset was limited to the first oscillating bump. The resetting effect after the rebound depolarization is particularly evident when multiple traces are overlaid and averaged (Figure 2B and 3A, black lines, bottom traces). Before the stimulus artifact, the subthreshold oscillations (due to phase-indepent nature of the stimulus trigger) are out of phase and the average is, therefore, deprived of oscillatory behavior. However, after the rebound depolarization the subthreshold oscillations of multiple traces are locked in phase, resulting in a prominent sinusoidal wave when averaged (Figure 2B). With a pronounced release of GABA, the IO neuron will be isolated from the network for a longer period, and this can be measured as the time needed for the phase-lock to fade away. In order to measure the reset accuracy and the decay of the phase-lock, we performed cross-correlations between all the possible combinations of pairs of traces of the same cell (a total of 36 traces) using a running window approach (see materials and methods). All analyzed cells showed the highest phase-match during the IPSPs (i.e. when the effect of GABA is maximal), followed by the rebound depolarization (namely the first peak of the subthreshold oscillation). Hereafter, the phase-match of the oscillation begins to deteriorate until the normalized cross-correlation value almost reaches zero. Figure 4A represents an example cell. The protocol was repeated 36 times in order to stimulate the cell at different random phase-points of its SSTO. All the traces from the same cell are overlaid in figure 4A (top). When the stimulation is given (red arrowhead) the neuron always responds with an IPSP, regardless the phase of the oscillation. A new oscillation is then initiated and its phase is reset in every repetition. The oscillation’s phase induced by the IPSP drifts with time and after two cycles of oscillation it is shifted. The correlation index between all the combinations of pairs of repetitions is averaged and shown in figure 4A (bottom). Then, we wanted to measure the decay time of the phase-lock, because this would reflect the duration of the action of the GABAergic activation. Therefore we fitted the correlation indexes with a single exponential function (figure 4B, red line) and we extracted the decay constant for each recorded neuron. We then plotted the durations of the IPSP vs the decay constants of the phase-match deterioration process of all the neurons (Figure 4B, exponential fit: r^2^ = 0.83, n = 11). We conclude that a longer inhibition comes together with a longer phase-lock in the oscillations and that both these phenomena possibly depend upon the amount of GABA released. Unfortunately, we could not explore this relationship in the presence of the GABA_A_R blocker, because SSTO cells often lose their oscillation profile before the DNDS exerts its complete effect.

Overall, a short burst of stimulation of the nucleo-olivary pathway induces a prominent long-latency inhibition in olivary neurons. The IPSP resets the sinusoidal oscillations via the post inhibitory rebound depolarization and determines the phase of the following SSTOs.

## Discussion

### Cerebellar Control of the Inferior Olive

Due to the anatomical position of the IO, which is difficult to approach, its network properties have mainly been investigated by indirect measurements such as complex spikes activity in the cerebellar cortex [2], [12], [37], [38] or by *in vitro* experiments [20], [25]. Our work shows for the first time, *in vivo*, CN-evoked IPSPs in olivary neurons and their dependency on the activation of local GABA_A_ receptors. Anatomical observations [3], [38]–[41] have demonstrated the presence of inhibitory projections from the CN to the IO, and Devor et al. [25] showed that GABA_A_ receptors can be activated on the soma and dendrites of IO neurons by puffing the GABA on different parts of the recorded neuron. The study of Best and Regehr [24] revealed that the release of GABA at CN to IO synapse is asynchronous. However, their olivary IPSPs/IPSCs were evoked *in vitro* via peri-olivary stimulation and by using an internal solution with high chloride concentration (to obtain a chloride reversal potential of −20 mV or 0 mV which amplifies the GABA_A_ mediated response). In contrast, our experimental approach combines the time resolution of the whole-cell recordings with the advantages of having an *in vivo* preparation, where the network and the synaptic connections are intact [7]. The stimulation protocol that we used was set coherently with the high-frequency input that CN neurons receive from PC and that was shown to elicit a rebound depolarization with increased firing frequency in CN neurons [42]. This increased firing activity in the CN has been shown to play a prominent role in processing and storage of information regarding motor coordination such as the conditioning response in classical conditioning [43]–[46]. The connections between the CN and IO are composed by a direct inhibitory nucleo-olivary pathway and by a disynaptic excitatory loop passing through the MDJ [41]. The stimulation of the CN can activate both pathways and consequently, based on pathway length, might be expected to elicit an EPSP in olivary cells after the occurrence of the IPSP. However, this sequence was never observed in our experiments (also see Ruigrok and Voogd, [7]); when an EPSP was evoked it always preceded the IPSP. This phenomenon can be explained by the fact that somatic GABA_A_ receptors of olivary neurons have slow activation kinetics, due to their slow-activating subunit composition (α_3_β_2/3_γ_2_, Devor et al., [25]), and that the release of neurotransmitter at the DC-IO synapse is asynchronous (Best and Regehr, [24]). This explains why the disynaptic EPSP can outpace the monosynaptic IPSP before the full-blown GABAergic shunting prevents any further electrical signaling. This hypothesis is also in line with the double stimulation experiments, which show how IO neurons undergo a long period of about 350 ms in which no excitatory input can be processed during the inhibitory phase of the response.

To confirm that the response we observed is due to direct activation of synapses that are directly connected to the recorded neuron rather then by an indirect activation through gap junctions as proposed by Ruigrok and Voogd [7], we performed a set of experiments in mice lacking the gap junction protein Connexin36. In these knock-out mice, we did not observe any difference in the sequence of the responses compared to that observed in the wild type littermates. The neuronal coupling between olivary neurons is, therefore, not required for the sequence and duration of the hyperpolarization indicating that they most likely reflect synaptic activation of the primary neuron.

Our experiments demonstrate that CN activation exerts a powerful inhibition of the IO and that all olivary neurons undergo a long-lasting silence. The hyperpolarization we observed is two times longer than that previously described in the cat by Ruigrok and Voogd [7]. This discrepancy can be attributed to either the difference in animal model, stimulation site or anesthetics. Our olivary neurons express different subthreshold profiles [22] which influence the response to CN activation. However, it’s still unclear whether the LTO and SSTO neurons reflect two different populations of IO cells or simply two different activity states of the same olivary neuron. In principle, the length of the inhibition was dependent on the state of the membrane potential. The relationship between the subthreshold profile and the length of the inhibition allow the system to influence the pattern generator (i.e. oscillations) very efficiently in a discrete temporal manner [47], [48]. After the strong inhibition, SSTO neurons show an intriguing rebound depolarization that is followed by a re-initiation of the sinusoidal subthreshold oscillation as shown in Figure 2B. It is noteworthy that the chances to observe a rebound depolarization are reduced in SSTO neurons after 20 minutes of DNDS perfusion (Table 2). This is probably due to the reduced GABA_A_-mediated hyperpolarization caused by the DNDS, which prevents the cell to generate the rebound depolarization. When the rebound depolarization is abolished then the oscillation is not re-initiated and there is no phase-locked oscillation. In this situation the subthreshold oscillation often reappears spontaneously after a while. Overlays of many olivary responses revealed the temporal accuracy of the oscillations and the speed/time at which oscillations can shift their phase after the stimulation of the CN. The phase-lock and decay in phase-match of the oscillation, studied here at a single cell level, are proportional to the duration of the GABAergic inhibition: short IPSPs are followed by a short decay in phase-match, whereas long IPSP is followed by much slower decay process. The duration of the GABAergic inhibition (i.e. hyperpolarization and shunting) is controlling the temporal accuracy of the oscillation. Our interpretation is that the asynchronous release of GABA [24] determines two different features of the GABAergic response: the first is the IPSP and the second is the phase-lock of the newly generated sinusoidal oscillation. The phase lock is only temporary and fades away within the following 800 ms. Since the asynchronous nature of GABA release at CN to IO synapse modulates the long-lasting IPSP according to the amount of neurotransmitter released [24], we hypothesize that a longer IPSP is caused by the activation of more CN-IO fibers. A more abundant asynchronous long-lasting release of neurotransmitter, however, would not only affect the duration of the IPSP itself, but probably will also have lingering effects on subsequent oscillations, which will be reflected in the duration of the phase-lock of the sinusoidal oscillations. These two features probably underlie the two roles of the IO, the former being the gating (caused by the IPSP) and the latter being the generation of temporal patterns (a longer phase-lock in the oscillations might indicate a reduced influence of the surrounding network).

Therefore, GABAergic input from the CN not only blocks temporarily all electrical signals, but it is also involved in the resetting of the temporal pattern generator (i.e. the sinusoidal subthreshold oscillation) and its temporal precision in the following cycles. Our results show for the first time how the IO is controlled by the feedback of the nucleo-olivary pathway: the activation of the CN elicits an IPSP which actively suppresses the instructive signal of the IO for approximately the length of one oscillation cycle (i.e. gating mechanism), and simultaneously resets the phase of the oscillation itself. This feedback gating mechanism corresponds with the neuronal correlate for motor learning proposed by Andersson [2]. In their model, the CN feedback to the IO is important for blocking the teaching signal when motor learning is already optimized and for extinction of the motor task when it is not relevant anymore [10], [37], [49]–[53].

## Materials and Methods

C57BL/6 male mice were imported from Harlan and housed at Erasmus MC in a 12-hour light-dark regime. Food and water were provided ad libitum. All animal procedures were in accordance with the guidelines of the Dutch Ethical Committee (DEC) at Erasmus Medical Center and the present study has been approved by the Institutional Animal Care and Use Committee (IACUC) of the Erasmus MC.

### Stimulus Electrode Placement

The animals were anesthetized with a mixture of ketamine and xylazine (65 and 10 mg/kg i.p), and body temperature was maintained at 37°C with the use of an anal thermosensor and a heating pad (FHC, Bowdoinham, ME). The occipital region of the skull was cleaned and a small opening was made in the occipital bone. Extracellular pipettes filled with 3 M K-Acetate were placed in the Interpositus Nucleus of the CN using stereotaxic coordinates. Extracellular signals were amplified with a CyberAmp 380 (Axon Instruments, Foster City, CA) and spiking patterns (irregular firing pattern below 10 Hz) were used to confirm the correct location. The extracellular pipette was removed and replaced by a custom-made bipolar epoxy-insulated tungsten electrode (impedance ∼300 kΩ) that was carefully lowered to the same position in the CN, either in the anterior or posterior interpositus nucleus. The recorded neurons in the olive where located in subnuclei receiving these projections, namely the caudolateral part of the Dorsal Accessory Olive (DAO) and in the rostral and central part of the Medial Accessory Olive (MAO) respectively. This is in accordance with the general topography of nucleo-olivary connections as they have been established in the rat [4]. The stimulus electrode was then glued to the occipital bone of the mouse. Misplacement of the stimulus electrode resulted in either a lack of olivary response or in a short-delayed response evoked by antidromical activation of collaterals of the climbing fibre. These fast responses were comparable with the ones previously described by Llinas and Yarom [14]: they are characterized by constant and short stimulation latency (less then 4 ms). Furthermore, the antidromic activation of the climbing fibre collaterals always elicits an action potential in olivary neurons (i.e. no failures). We exclude recordings obtained by misplaced stimulation electrodes and antidromically activated neurons. The stimulations protocol consisted of short high frequency bipolar stimulations (3 pulses; pulse frequency: 300 Hz; pulse duration: 0.2–0.3 ms; pulse intensity: <0.1 mA). Under our experimental conditions, we were not able to induce inhibitory or excitatory responses in olivary neurons by long low-frequency bipolar stimulations of the CN (3, 20 or 40 pulses; pulse frequency: 20 Hz; pulse duration: 0.2–0.3 ms; pulse intensity: <0.1 mA, as described by Best and Regher., 2009). Instead, our stimulus protocol was similar to the one described by [7] and represents the fast rebound burst spiking of CN neurons after a strong inhibition [42], [54]. At the end of the experiment, a lesion was made with high-intensity current injection to confirm the position of the tungsten electrode (Figure 1C).

### *In Vivo* Whole-Cell Recordings

To perform stable *in vivo* recordings in the IO, the mouse was placed in supine position and the head was restrained. In this way a ventral approach of the medulla oblongata was performed and the dura mater was removed to expose the ventral surface of the brainstem (Khosrovani et al. [22]). Whole-cell recordings were performed with borosilicate pipettes (with filament; outer diameter: 1.5 mm; inner diameter: 0.86 mm; Sutter, California, USA) filled with 4 mM NaCl, 3.48 mM MgCl_2_, 9 mM KCl, 10 mM KOH, 120 mM K-gluconate, 10 mM Hepes, 29 mM sucrose, 4 mM Na_2_ATP, and 0.4 mM Na_3_GTP with pH 7.2 and osmolarity at 290–310 mOsm/kg. In the pharmacological experiments, 5 mM 4,4-dinitrostilbene-2,2′-disulfonate (DNDS; GABA_A_ receptors blocker [29], [30] was added to the pipette solutions and pH and osmolarity corrections were made. Electrode resistances ranged between 4–8 MΩ and the junction potential was approximately −8 mV; membrane potentials were corrected for this value. Current clamp recordings were amplified with a Multiclamp 700B (Axon Instruments, Foster City, CA), filtered at 10 kHz and digitized at 20 kHz with a Digidata 1322A (Axon Instruments). Membrane passive properties were determined as in Khosrovani et al. [22]; resting membrane potential of SSTO neurons refers to the mean value of the membrane potential between the peak and the trough. It has been shown that NMDA receptors are fundamental for the generation of sinusoidal oscillations [19] and for this reason the use of Ketamine/Xylazine in our experimental approach could be arguable. However, Khosrovani et al. [22] showed how the use of different anesthetics (Medetomidine-Midazolam-Fentanyl) gives result comparable to the ones observed under Ketamine/Xylazine anesthesia. Moreover, the subthreshold oscillations recorded in our experiments (both LTO and SSTO) are in line with the ones observed *in vitro* [14], [17], [26], and also *in vivo* using a different anesthetic, halothane [21]. In conclusion, we used Ketamine/Xylazine anesthesia because it was necessary and because, to our knowledge, it does not show any specific effect in the IO.

### Data Analyses and Statistics

Data analyses were performed on neurons with resting membrane potentials negative to −45 mV and stable access and stable membrane resistances throughout the recording. Data analyses were performed with Clampfit software 9.2 (Axon Instruments, Foster City, CA).

Stimulation protocols were repeated at least 36 times for each neuron. In between the stimulation sessions the neurons’ activity was monitored to discard those elements whose input resistance varied more than 20% of the initial value during the recording. In olivary neurons, excitatory postsynaptic potentials (EPSP) and inhibitory postsynaptic potentials (IPSP) were all evoked by CN stimulation. On some occasions the evoked EPSP was strong enough to elicit a sodium spike followed by afterdepolarization (ADP, as described by Llinas and Yarom [14] and Ruigrok and Voogd [7]), but in most cases only a subthreshold response was observed. The percentage of successful responses was calculated by counting the number of responses and dividing the total by the total number of stimulations. The response delay was determined by measuring the latency between the last stimulation and the start of the evoked depolarization. Of all evoked IPSPs, the peak value of the hyperpolarization and the surface area generated by the hyperpolarized membrane potential were determined. This surface area was measured between the starting point and endpoint of the IPSP by an integration algorithm implemented in Clampfit 10.2. The starting point and endpoint of IPSPs were determined by using a threshold potential of −2 mV negative to the resting membrane potential. If there was any subthreshold sinusoidal oscillation the resting membrane potential (Vm) was determined from the (low-pass) readout of the baseline potential. The rebound depolarization at the end of the long latency IPSP was identified as an upward deflection of the membrane potential that was at least 2 mV greater than the value of the membrane potential preceding the IPSP. In order to investigate the impact of the subthreshold oscillation on the CN induced response, we subdivided the olivary cells into two groups on the basis of their subthreshold activity: the spontaneous 3 – to 12 Hz sinusoidal subthreshold oscillating cells (SSTO) and the spontaneous 1 – to 3 Hz low-threshold Ca^2+^ oscillating cells (LTO). The use of the frequencies of subthreshold oscillations to categorize different groups of olivary cell has been justified by cluster analysis in Khosrovani et al. [22]. Comparison between groups and conditions were made by using two-tailed Student’s *t*-test, and p values were adjusted by using the Bonferroni correction method. All the values express average ± standard deviation (SD). Cross-correlograms were performed in the Matlab (The MathWorks, Inc., Natick, MA) environment using a custom-made function. The aim of the analysis was to measure the kinetics of the CN-activation induced phase-lock and the developing phase variance of the sinusoidal subthreshold oscillations. For each recorded SSTO neuron, 36 randomly-started stimulations were analyzed, and cross-correlograms were computed between all the possible combinations of couples of stimulations (i.e., 36*36 = 1296 combinations) using a “running window” of 100 ms that shifted along the full length of the recordings by 50 ms steps. The cross-correlograms computed between all the possible combinations of pairs of recordings were then averaged and fitted with a single exponential function to extract the time constant from all of the 11 SSTO neurons analyzed (Figure 4). The decay time constants of the cross correlograms were plotted against the length of the hyperpolarization, and this data set was fitted using a single exponential function.

## Supporting Information

Figure S1 **Double stimulation experiments.** An LTO neuron responding with a short latency EPSP is stimulated twice at different time intervals (first trace single stimulation, then 25, 175, 250, 350 ms intervals respectively, stimulation artifacts are indicated by red arrow heads). A second EPSP is evoked when the time interval is at least 350 ms (indicated by the asterisk). Every trace represents the average of 12 repetitions.
(TIF)

Figure S2 **Experiment performed with 15mM DNDS.** Wild type SSTO cell (top trace) responding to CN stimulation, then (middle trace) the response of the same cell to CN stimulation in reduced after 20 minutes of dialysis with DNDS 15 mM, bottom trace represents the averages at the beginning of the experiment (black) and after 20 minutes of DNDS 15 mM (red). Even with high concentration of DNDS the IPSP is not completely abolished.
(TIF)
