# Supplementary figures and images for: Properties of the Nucleo-Olivary Pathway: An In Vivo Whole-Cell Patch Clamp Study
Source: PLoS One. 2012 Sep 27;7(9):e46360. doi: 10.1371/journal.pone.0046360 (PMC3459892; doi:10.1371/journal.pone.0046360)

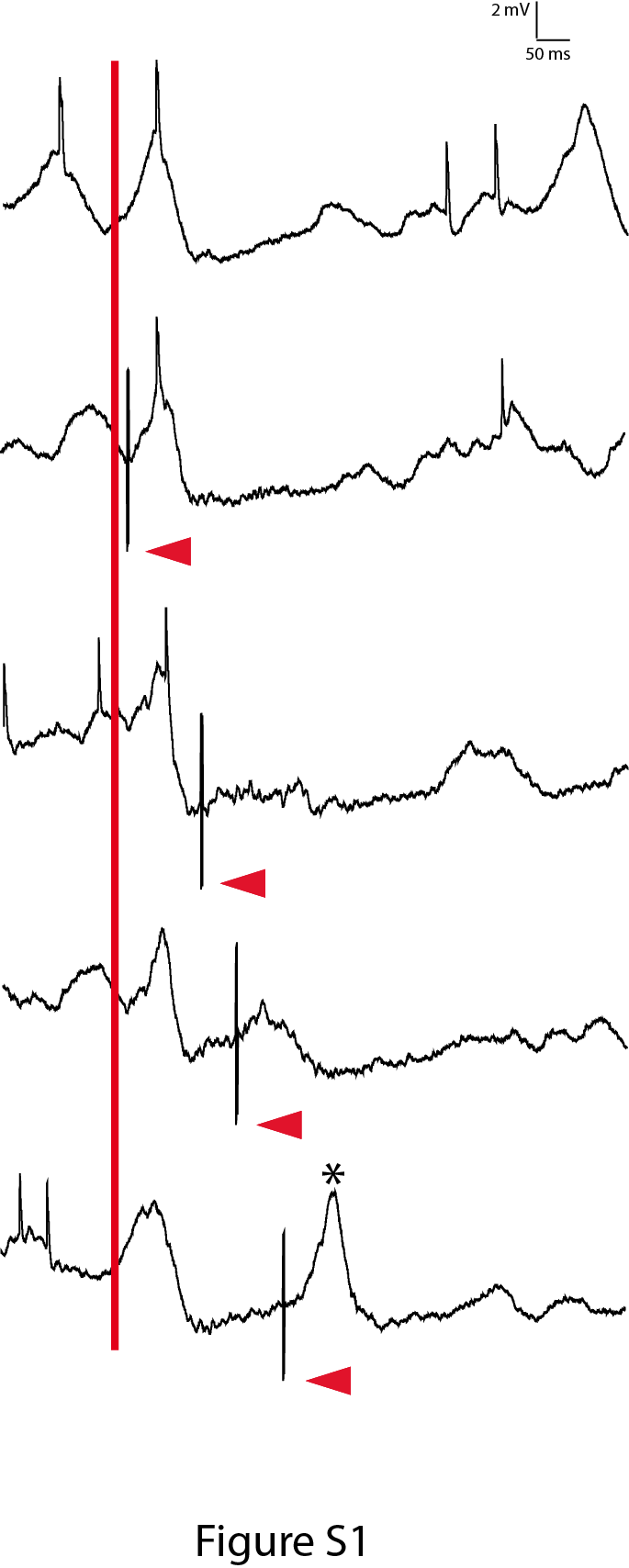

Supplement: Figure S1 — Double stimulation experiments. An LTO neuron responding with a short latency EPSP is stimulated twice at different time intervals (first trace single stimulation, then 25, 175, 250, 350 ms intervals respectively, stimulation artifacts are indicated by red arrow heads). A second EPSP is evoked when the time interval is at least 350 ms (indicated by the asterisk). Every trace represents the average of 12 repetitions. (TIF) [file pone.0046360.s001.tif]

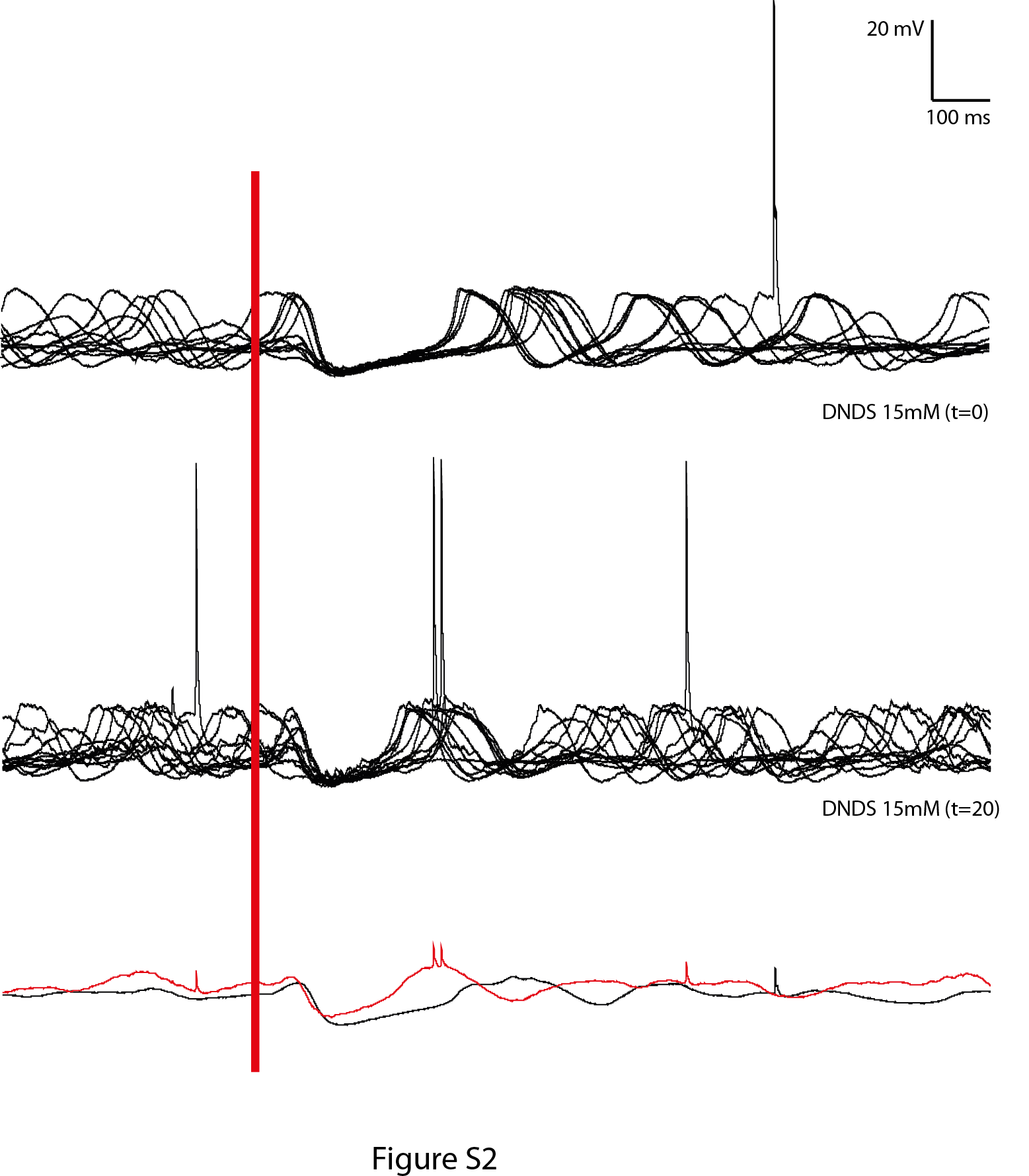

Supplement: Figure S2 — Experiment performed with 15mM DNDS. Wild type SSTO cell (top trace) responding to CN stimulation, then (middle trace) the response of the same cell to CN stimulation in reduced after 20 minutes of dialysis with DNDS 15 mM, bottom trace represents the averages at the beginning of the experiment (black) and after 20 minutes of DNDS 15 mM (red). Even with high concentration of DNDS the IPSP is not completely abolished. (TIF) [file pone.0046360.s002.tif]
